# Retraction: Defining pathogenic bacterial species in the genomic era

**DOI:** 10.3389/fmicb.2026.1934031

**Published:** 2026-07-20

**Authors:** Frontiers Editorial Office

The journal retracts 17 January 2011 the article cited above.

Following publication, concerns were raised regarding several scientific and editorial issues within the article:

- Multiple Duplications: Multiple instances of image and content duplication were identified within the figures.
- Uncited Sources: A source for one of the key figures was left uncited.
- Irrelevant Citations: Irrelevant or mismatched citations were found associated with the published figures.

An investigation was conducted in accordance with Frontiers' policies and the Committee on Publication Ethics (COPE) guidelines. The authors were contacted and requested to provide clarifications and submit a formal Correction to resolve these issues. However, the authors remained unresponsive to multiple communications and Correction requests.

Due to the unresolved concerns regarding image integrity, attribution, and citation accuracy, as well as the authors' lack of response, the journal has retracted the article.

This retraction was approved by the Chief Editors of *Frontiers in Microbiology* and the Chief Executive Editor of Frontiers. The authors do not agree to this retraction.

Frontiers would like to thank Dr. Elisabeth Bik for contacting the journal regarding the published article.

